# Evaluation of the obturator foramen as a sex assessment trait

**DOI:** 10.1007/s12024-022-00514-0

**Published:** 2022-08-12

**Authors:** Samuel R. Rennie, Constantine Eliopoulos, Silvia Gonzalez

**Affiliations:** 1 Department of Life and Environmental Sciences, Faculty of Science and Technology, Bournemouth University, Poole, BH12 5BB UK; 2 School of Biological and Environmental Sciences, Liverpool John Moores University, Liverpool, L3 3AF UK

**Keywords:** Sexual dimorphism, Sex determination, Pelvis, Obturator foramen, Forensic anthropology

## Abstract

Correctly assessing sex from skeletal remains is one of the main elements of creating a biological profile. Many traits allow for this, the obturator foramen being one. However, research on its accuracy has provided mixed results. This study examines the obturator foramen using a 5-point grading scale to assess the degree of sexual dimorphism in four known age and sex skeletal collections from the UK and South Africa. Overall, sexual dimorphism was found in the obturator foramen when using the new scoring system; however, accuracies for correct sex classification ranged from ~ 46 to ~ 75%. Considering its wide range in accuracy rates across the four samples and difficulty in identifying the subtle changes in morphology, the obturator foramen should only be used as part of a multifactorial assessment of sex.

## Introduction

Sex determination, whether for forensic or bioarchaeological purposes, is one of the most critical aspects of creating a biological profile [1, 2]. Correct classification can lead to a positive identification of an individual or a more accurate portrayal of a past population. Many studies have been conducted on the human skeleton; however, the *os coxae* s considered to be the most sexually dimorphic bony element [3, 4] because of the morphological differences between the sexes that are related to parturition and locomotion [5]. Because of these known differences, the *os coxae* has been studied extensively both quantitatively [6, 7] and qualitatively [8, 9].

Holobinko [10] stated that cranial traits should be secondary if the pelvis is present due to the pelvis having a higher degree of sexual dimorphism than other skeletal regions [11, 12]. One of the most well-established methods for visually sexing the skull achieved accuracies ranging from 84 to 88% [13], while accuracies for the *os pubis* achieved rates as high as 96% [8]. Therefore, it is understandable that the pelvis is preferred over the cranium [14].

The most well-known technique for morphologically assessing the *os coxae* is the Phenice method [8]. Over the years, it has been tested extensively on various populations and has become a standard in sex determination [15–18]. Klales et al*.* [19] re-evaluated the Phenice trait and expanded the descriptions of how each of the three traits is expressed. The revised version of this Phenice method achieved a correct independent classification of 86.2%.

**Fig. 1:**
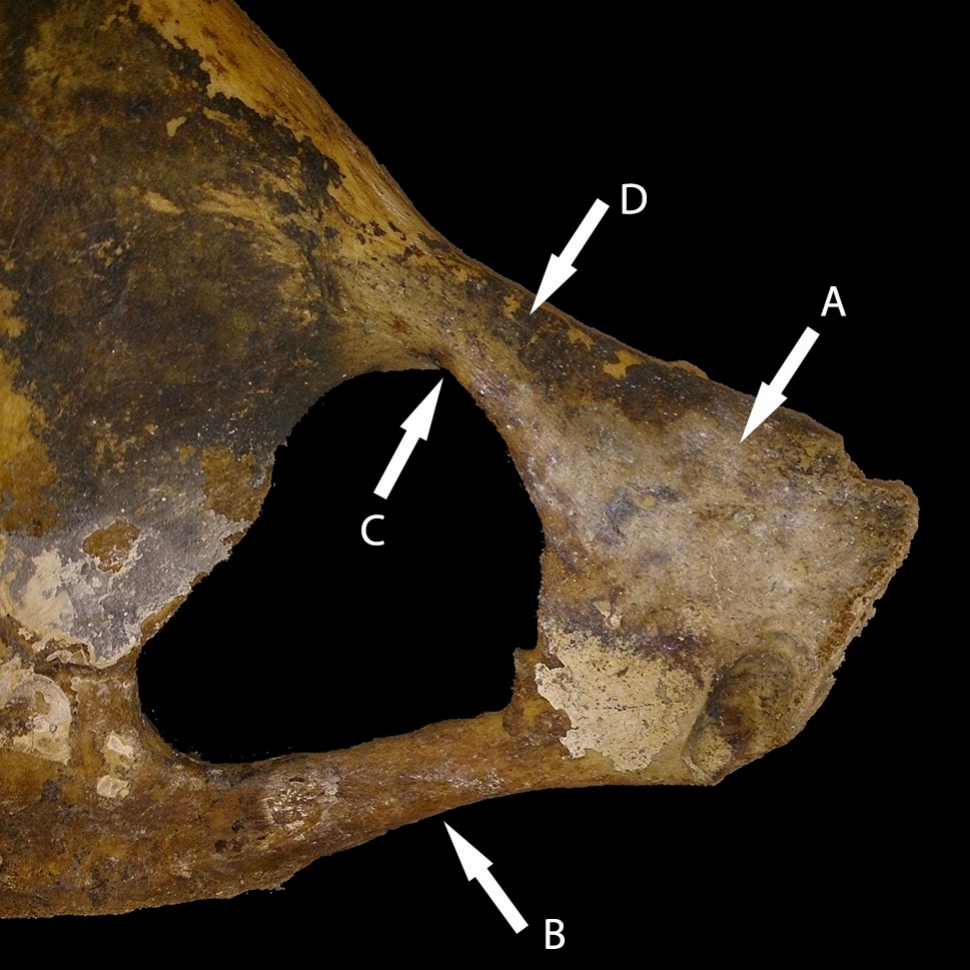
CAS 2070 Female, 35 years old, Spitalfields, London, UK (Natural History Museum, UK) (image taken by author). Dorsal view of the left anterior pelvis. **A** *Os pubis*, **B** ischiopubic ramus, **C** obturator groove, and **D** iliopubic ramus

The obturator foramen is formed by the interior borders of the pubis and ischium. The obturator foramen houses several soft tissue structures, such as the obturator membrane, which allows the obturator artery, vein, and nerve to pass through the canal. Superiorly to the membrane lies the obturator groove to which the vessels pass. The obturator membrane attaches to two tubercles on the foramen, the posterior and anterior obturator tubercle. The posterior tubercle is located along the medial border of the ischium, while the anterior tubercle lies in the superior ramus of the pubis (Fig. 1).

Sex differences have been described for the obturator foramen by many authors [20–22]. The differences as they have been reported are related to size, with the foramen being larger in males and shape, with males having an oval foramen while females a more triangular one. These descriptions have not changed in over 200 years of medical/anatomical research since they were first published in the late eighteenth century by Ackermann [23].

Considering this long history, very little work has been undertaken into why the obturator foramen is sexually dimorphic, and there have been little to no updates on the classification descriptions.

The Workshop for European Anthropologists [24] recommended a 5-point grading system that could be used to score the expression of the obturator foramen along with other morphological features. Rogers and Saunders [25] used many morphological sexing traits to assess which individual traits and combinations diagnose sex with the highest accuracy. They found that by combining the obturator foramen with the ventral arc or the true pelvis shape, the accuracy was as high as 98%. This ranking of the obturator foramen within the top f5 traits used for sex determination contradicts results by St. Hoyme [26], who stated that the foramen had very little value when used.

Eliopoulos [27] followed the general descriptions of “oval” and “triangle” when assessing the obturator foramen on the Athens Collection, which he created as part of his doctoral thesis. He found that overall accuracy of 43.8% was achieved, with a heavy sex bias between classification accuracy (male = 67.6%; female = 14.0%).

Metric analysis has also been conducted to help create a more objective approach to assessing the shape of the foramen. Quantitative analysis was first attempted by Martin [28], who used four landmark points to take measurements from and create an index for sex differences. Martin [28] found accuracies of ~ 65%.

With the advancement of statistical tests, Bierry et al. [29] applied an Elliptical Fourier analysis to assess the obturator foramen. They used computerised tomography (CT) images from the virtual collection at the University Hospital in Strasbourg. The application of elliptical Fourier analysis with the addition of a discriminant function analysis, reached an overall accuracy of 84.6%.

The present study aims to assess whether the obturator foramen can be used reliably in the sex determination of human skeletal remains.

## Materials and methods

Samples from different geographic/ancestral backgrounds were observed. The material used for this study is derived from four skeletal collections: Christ Church, Spitalfields Collection housed at the Natural History Museum (UK) [30–32], the Pretoria Bone Collection housed at the University of Pretoria (Republic of South Africa) [33], the Raymond A. Dart Collection of Human Skeletons housed at the University of the Witswatersrand (RSA) [34], and the Kirsten Collection housed at Stellenbosch University (RSA) (Table 1). For individuals to be selected for analysis, only those with a partial or complete pelvis aged 18 years and above were chosen. The lower age limit of 18 years was used as many authors define this as the start of adulthood [35]. Following these selection criteria, an overall sample size of 741 human *os coxae* were examined.

**Table 1:** Demographic characteristics of the study samples

Sample		*N*	Mean age (years)	Min–max (years)
Christ Church, Spitalfields				
	Male	69	57.29	22–91
	Female	69	58.43	23–89
	*Total*	*138*	*57.86*	*22–91*
South African White				
	Male	95	63.16	28–94
	Female	98	67.51	19–88
	*Total*	*193*	*65.37*	*19–94*
South African Black				
	Male	108	45.44	20–86
	Female	97	45.05	20–80
	*Total*	*205*	*45.26*	*20–86*
South African Coloured				
	Male	102	53.34	19–101
	Female	103	46.17	18–89
	*Total*	*205*	*49.74*	*18–101*

The Spitalfields Coffin-Plate collection is an eighteenth to nineteenth century British skeletal sample that was excavated from the church grounds and vaults in 1984 [30–32]. A total of 968 individuals were originally exhumed, however, only 387 were associated with coffin plates that contained the individual’s name, age, and year of death. Following this, archival research of parish records helped confirm the age and sex of these individuals. Due to the varying degrees of preservation, a total of 138 adult *os coxae* were physically examined, with an age range of 22–91 years old.

The three South African assemblages (South African White, Black, and Coloured) are from modern day cadaver-based human skeletal collections; thus, sex and age are determined from physical examination before dissection and medical records associated with each individual and as a result, they are considered to be accurate. An overall total of 603 South African adult *os coxae* were physically examined (South African White = 193, South African Black = 205, South African Coloured = 205), ranging between 19 and 101 years old.

South African Black refers to individuals from the two main groups: Nguni and Sotho-Tswana. The former includes individuals who are Zulu, Xhosa, Ndebele, and Swazi; the latter includes Southern, Northern and Western Sotho [36]. The term South African Coloured refers to an individual from a highly diverse social group with a wide range of phenotypic variability [37]. The group consists of people of mixed ancestry, from descendants of the Khoesan subgroup and enslaved people later brought to South Africa from Malaysia and Indonesia by the Veerenigde Oost-Indische Compagnie [36, 37]. A large contribution to the modern-day coloured South African group is from various other groups brought to the Cape colony by the European settlers. Some contributing populations include Indonesia, Malaysia, and India [38, 39]. The term “South African Coloured” is the accepted term used in South Africa. Ethical approval and all relevant permits were obtained by each institution prior to data collection.

**Fig. 2:**
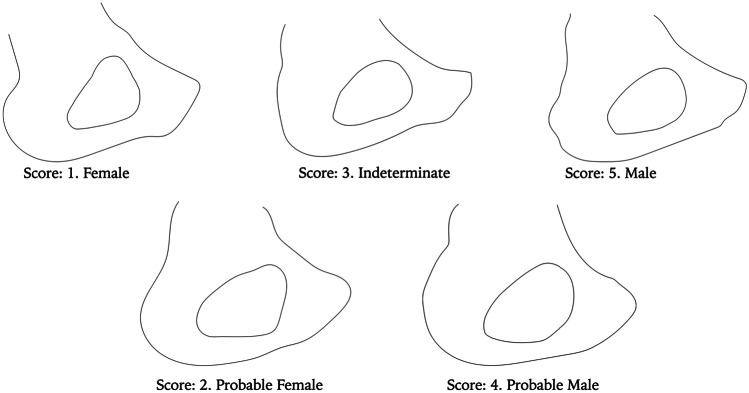
Morphological scoring of the obturator foramen (dorsal view) ranging from female (score 1) to male (score 5)

**Fig. 3:**
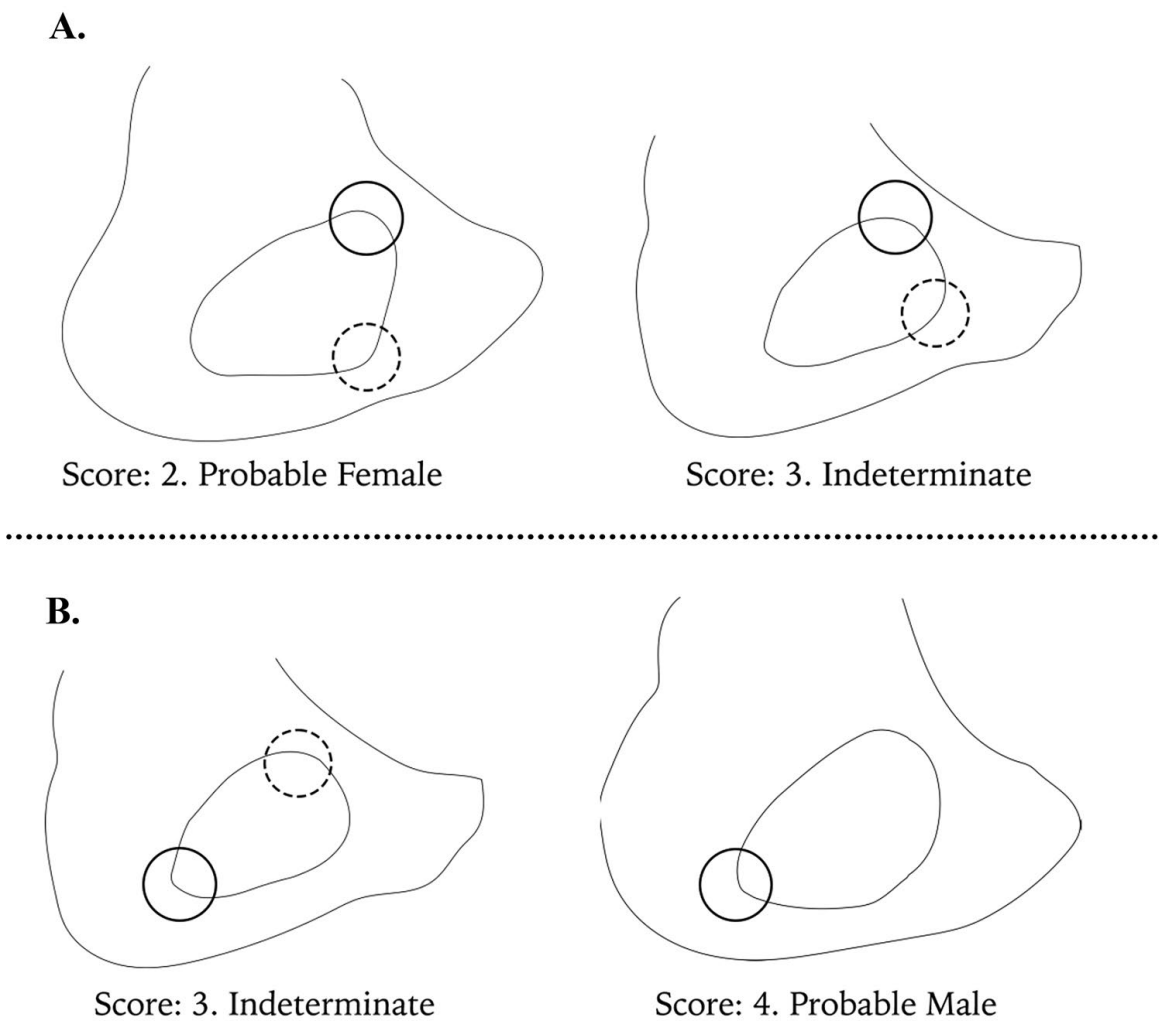
Illustration of the landmarks used **A** anterior superior angle (solid circle) and anterior inferior angle (dashed circle) are more defined/angular in score 2 than in score 3. **B** Posterior inferior angle (solid circle) is more defined in score 3 than in score 4. In score 3, the anterior superior angle (dashed circle) is present whilst in score 4, it is not

The following descriptions and images were created for a 5-point ordinal scale to assess the overall expression of sex (Figs. 2 and 3). This method was applied to each *os coxae* blind, meaning the age and sex of the individual were not known when scoring the obturator foramen. This was to counter any bias in the application of the scoring system. The left *os coxae* were physically examined and if it was unavailable, the right was analysed in its place.

1. Overall shape is triangular. Clear angular definitions in all three areas. Both superior and inferior borders become straight. *Female*
2. Inferior border becomes straight. Superior border becomes more straight but still retains a small amount of concavity. Anterior inferior and anterior superior portion becomes angular (Fig. 3a). *Probable Female*
3. Obturator foramen shows a more prominent posterior inferior angle, with the anterior superior portion starting to become angular (Fig. 3b). *Indeterminate*
4. Obturator foramen is oval; however, the posterior inferior portion is slightly angular (Fig. 3b). *Probable Male*
5. Oval in shape and appearance. All borders are smooth. *Male*

Classification rates (including sex biases in the scoring method) and intra/inter-observer errors will be analysed. The Kappa Cohen’s test for repeatability following Landis and Koch’s [40] definitions will be used to calculate observer error. A randomised subsample (*n* = 73) was created to perform the intra/inter-observer error. The intra-observer analysis was performed by the first author and was conducted 2 weeks after the initial data were collected. Two observers were chosen to conduct the inter-observer analysis. The first observer (Observer 1) holds an M.Sc. in Anatomy and has more than 5 years of experience assisting in forensic anthropology casework. The second observer (Observer 2) holds a PhD in Biological Anthropology and has more than 5 years of experience in the skeletal analysis of archaeological skeletal remains. Both conducted the inter-observer error with no assistance, only the descriptions of the scoring method.

## Results

All four samples showed a significant difference in the obturator foramen scores between the sexes suggesting that the obturator foramen is sexually dimorphic (Black South African: *U* = 7541.00, *p* < 0.001; Coloured South African: *U* = 6450.50, *p* < 0.01; White South African: *U* = 7332.00, *p* < 0.001; Spitalfields, UK: *U* = 4051.50, *p* < 0.001).

**Fig. 4:**
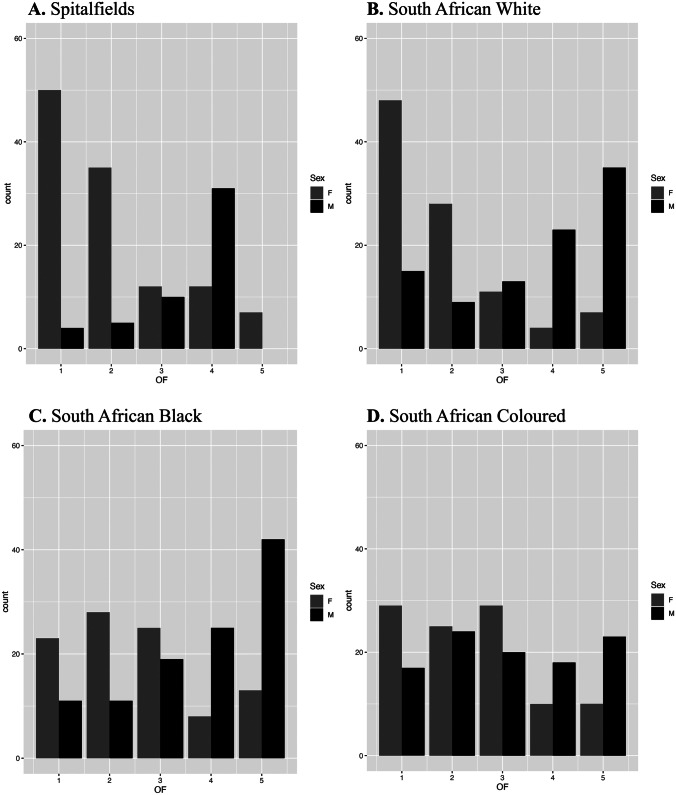
Frequency of obturator foramen scores. **A** Spitalfields, **B** South African White, **C** South African Black, and **D** South African Coloured

The spread of scores presented in Fig. 4 illustrates this sexual dimorphism, as a bimodal distribution is evident, especially in the Spitalfields and South African White samples.

Since previous research has suggested that the obturator foramen is sexually dimorphic [20–22, 24, 29], classification rates were calculated for each sample. Because the method has a 5-point ordinal scoring design, a correct female classification was made if a score of “1” or “2” was made, and for males, it was a score of “4” or “5”. Intermediate scores of “3” were classed as incorrect. Results can be seen in Table 2.

**Table 2:** Classification accuracies for each sample

**Sample**	Male correct (%)	Female correct (%)	**Overall correct (%)**
South African (Black)	62.0	52.6	**57.3**
South African (Coloured)	40.1	52.4	**46.25**
South African (White)	61.0	77.6	**69.3**
Spitalfields, UK	82.6	68.1	**75.35**
*Overall*	*61.43*	*62.68*	***62.05***

Despite the fact that each sample showed sexual dimorphism, the ability of the obturator foramen to determine sex correctly differs greatly between each sample. The highest overall accuracy was from the Spitalfields, UK sample, which achieved 75.4%. The sample also had the highest male and female accuracy of 82.6% and 68.1%, respectively. A similar trend can be seen for the White South African and Black South African samples but with much lower male, female, and overall classification accuracies (Table 2). The lowest accuracy was seen in the Coloured South African sample. This had an overall sex accuracy of 46.3%, with 52.4% of females and 40.1% of males being correctly identified. In addition, this result is less than 50%; therefore, it would be more accurate to randomly assign sex than to use the obturator foramen for sex determination for this sample.

Using Landis and Koch’s [40] definitions of the Kappa values, the obturator foramen had a substantial agreement between the intra-observer scores (*k* = 0.676, *p* < 0.001). When performing the inter-observer error, the obturator foramen had a moderate agreement between observers with a much lower Kappa value (*k* = 0.529, *p* < 0.001).

## Discussion

The present study shows a significant difference in the scores between the sexes with a combined overall accuracy of 62.05%. When classification accuracy for each sample was calculated, it was found that there was a considerable difference between males and females, with a bias towards males in two of the four samples (Table 2). It is interesting to see that this bias seems to have no grounding in an individual's ancestry since male bias is seen in South African Black and Spitalfields samples and female bias in the South African Coloured and White samples. A difference was also found between the four samples tested. The most considerable difference in accuracy was seen in male samples between Spitalfields, UK, and the Coloured South African sample having a discrepancy of 40.5%. It is worth noting the difference in overall accuracy between the Spitalfields and South African White samples (75.35% and 69.3%, respectively), and the South African Black and Coloured samples (57.3% and 46.25%, respectively).

In terms of intra/inter-observer error, Eliopoulos [27] achieved an intra-observer agreement of 81.4% and an inter-observer agreement of 50.8%, which mimic what was found in the present study. The low inter-observer agreement found in this study (*k* = 0.529) causes issues when trying to implement new techniques; if they cannot be repeated accurately by other observers then the technique should not be used regardless of the accuracy it can produce.

Rogers and Saunders [25] found that the obturator foramen was the second most effective sex indicator for their Canadian sample, with accuracies as high as 93.8% which contradict the results found here. There are several reasons why there is a discrepancy. Firstly, the individuals from Canada may exhibit extreme morphologies for males and females for the obturator foramen. Secondly, they use descriptions made by St. Hoyme [26], where no visual diagrams can be found; therefore, trying to recreate their study regarding the obturator foramen is impossible. If the descriptions from Ferembach et al. [24] were the same as St. Hoyme [26], then caution should be taken as there are no visual diagrams for how to score this trait, and the only description for each score contains a maximum of three words. Fourth, because a new scoring method was created for this study, different criteria were used, which could account for some discrepancies between this study and Rogers and Saunders’ [25]. However, there is nearly a 20% difference in accuracy when comparing Rogers and Saunders’ [25] result with Spitalfields from this study. This study is more in agreement with the interpretation made by St. Hoyme [26] that the foramen has little value when considering its use for sex estimation despite it showing signs of sexual dimorphism. Accuracies did differ between populations in this study but averaged an overall accuracy of 62.05%, similar to Arsuaga and Carretero [41] (63.5% correct classification). Comparing the results to Beirry et al. [29], significant differences in accuracy rates are present. However, this can be explained by using Fourier analysis and Discriminant Function Analysis to categorise the oval (male) and triangular (female) expressions rather than relying on the naked eye. Ridgeway et al. [42] concluded after analysing 96 female pelves that the considerable amount of shape variation seen in the obturator foramen could be due to an individual’s height, noting that the greater the foramen area is, the taller the individual was. Ridgeway and colleagues [42] found no significant difference between shape and a person’s ancestry. Because an individual’s height was not considered in the present study, as it would have been largely estimated, unlike the known height in Ridgeway’s study, their conclusions could not be tested. However, a large amount of variation was seen not only between samples but within samples as well (Table 2 and Fig. 4), which coincides with the findings of Ridgeway et al*.* [42] that there is no link between shape and an individual’s race.

Waldron [43] and Stojanowski et al. [44] observed the preservation rate of skeletal elements from different archaeological assemblages and found that when focussing on the pelvic bones, the sacroiliac joint was preserved at a much higher frequency than the *os pubis*. The obturator foramen is not preserved when the break occurs along the inferior and superior ramus of the pubis. The low preservation of the *os pubis* in archaeological and forensic cases means this method is not usually performed.

The obturator foramen seems to provide variable success across the literature, with more literature stating its limited use in sex determination [26, 27, 41]. With this in mind, the results of the present study we can conclude that the obturator foramen should not be used to determine sex on its own as it is a poor discriminator. Arguments can be made to include it in a multifactorial assessment that includes other morphological traits of the pelvis. However, since most other traits outperform the obturator foramen, and the fact that it is prone to be non-assessable if the pelvis is fragmented, which happens frequently, we do not recommend its use for the assessment of sex.

## Key points

1. Applying obturator foramen morphology to assess sex in human skeletal remains has received varying degrees of success.
2. A new morphological scoring system explaining the sexual variation of the trait was applied to four known age and sex human skeletal populations.
3. Results from this study show accuracies ranging from 46.25%—75.35%
4. Due to this result and the obturator foramen’s variability, the trait should not be used to assess sex from human skeletal remains.
